# Anti-colorectal cancer effects of scutellarin revealed by genomic and proteomic analysis

**DOI:** 10.1186/s13020-020-00307-z

**Published:** 2020-03-26

**Authors:** Liu-Lin Xiong, Ruo-Lan Du, Lu-Lu Xue, Ya Jiang, Jin Huang, Li Chen, Jia Liu, Ting-Hua Wang

**Affiliations:** 1 Institute of Neurological Disease, Translational Neuroscience Center, West China Hospital, Sichuan University, Chengdu, 610041 China; 2 Animal Zoology Department, Institute of Neuroscience, Kunming Medical University, Kunming, 650000 Yunnan China; 3 Department of Anesthesiology, The Affiliated Hospital of Zunyi Medical University, Zunyi, 563000 Guizhou People’s Republic of China; 4 School of Pharmacy and Medical Sciences, Division of Health Sciences, University of South Australia, Adelaide, SA Australia

**Keywords:** Colorectal cancer, Scutellarin, Antitumor effect, Genomics, proteomics

## Abstract

**Background:**

Colorectal cancer, one of the most common digestive tumors with high mortality and morbidity worldwide, currently lacks effective therapies available to improve the prognosis. This study was aimed to investigate the potency of Scutellarin against colorectal cancers, and explore the related mechanism via genomic and proteomic analysis.

**Methods:**

Cell counting kit-8 assay was employed to detect the viability of HCT-116 and RKO cell lines treated with Scutellarin. The apoptosis of HCT-116 and RKO cells after Scutellarin administration was determined by TUNEL staining and Caspase 3/7 activity. Cell cycle was detected by flow cytometry analysis. The wound healing and transwell invasion test detected the role of Scutellarin in migration and invasion of HCT-116 and RKO cells. Meanwhile, the energy metabolism and growth of tumor tissues in vivo at day 28 were observed by PET-CT after Scutellarin administration with 50 mg/kg, 100 mg/kg and 300 mg/kg into 4-week-old nude mice. Blood routine and liver functions were also detected to evaluate the side effect of Scutellarin. Furthermore, the disease and function classifications which the differentially expressed genes and proteins involved after Scutellarin treatment were determined by genomic and proteomic analysis respectively.

**Results:**

The Scutellarin inhibited the migration and increased apoptosis of HCT-116 and RKO cell lines. Besides, Scutellarin treatment substantially decreased the growth and volume of colorectal tumors in nude mice without side effects on the blood routine and liver function. The differentially expressed genes in RKO cells after Scutellarin administration were mainly enriched in cell death and survival, organismal injury and abnormalities, and cancer. In addition, forty-seven upregulated and twenty-nine downregulated proteins were identified. Functional clustering analysis exhibited enriched biological processes, cellular components, molecular functions and related pathways of these proteins in cellular metabolic. Then protein–protein interactions analysis showed the regulatory relationship among these differentially expressed proteins.

**Conclusions:**

Taken together, the present findings revealed that Scutellarin exerted significant antitumor effect with no side effects in the blood and liver by regulating various important molecules in tumor proliferation, apoptosis and metastasis.

## Background

Colorectal cancer (CRC) is one of the leading causes of cancer-related mortality and with high morbidity [1]. In 2012, 1.4 million people worldwide were diagnosed with CRC and 6,93,900 died. [2]. CRC usually results from a combination of inherited susceptibility together with environmental factors [3]. Those whose first-degree relatives were diagnosed with CRC had a high lifetime risk of developing CRC [4, 5]. Numerous treatments for CRC have been extensively studied and applied. Screening programs could conduce to stratifying those patients tending to relapse, and personalized approaches may be designed to enhance the survival rate of postoperative CRC (PCRC) patients [6, 7]. Surgical treatment is primarily applied to 80% CRC patients without metastatic disease, but more than 40% patients in stage II or III may suffer from postoperative progression [8, 9]. In some cases with residual cancer, tumor size surged swiftly after operation [10]. Thus investigations for more effective therapies are of vital urgency.

Traditional Chinese Medicine (TCM) has been employed in cancer treatment for a long time. In view of serious side effects caused by radiation therapy and chemotherapy, more attention has been paid to TCM for the prevention and treatment of cancers worldwide. TCM treatment is beneficial for cancer patients in terms of modulating immunity, enhancing efficacy, reducing adverse effects and abrogating drug resistance, which represents a promising complementary approach in CRC prevention and treatment [11, 12]. Scutellarin (SCU), extracted from the dried Erigeron, is an active ingredient of flavonoid glucoside [13] and has been proved to have anti-inflammatory, anti-virus, anti-fibrotic, and immune regulation effect [14, 15]. In recent years, SCU has been found to exert anti-tumor effects on cancer cells, such as breast carcinoma, ovarian carcinoma and renal carcinoma [16–18]. Uprising evidence has exhibited the potentials of SCU for colorectal cancer treatment indicated by inhibiting angiogenesis, proliferation, invasion and metastasis of colorectal cancer cells, as well as inducing apoptosis [19, 20]. However, these reports mainly elaborated the efficacy of SCU and its related mechanisms from in vitro experiments verification. Herein, the anticancer activities of SCU in colorectal cancer were determined by in vitro and in vivo investigations, and on account for the complexity of cancer cells metastasis and our goal to successfully target it in cancer treatment, we attempted to analyze the preliminary molecular mechanisms of SCU underlying its anti-cancer efficacy via genomic and proteomic analysis.

## Materials and methods

### Cell culture

HCT-116 [ATCC number: CCL-247] colorectal carcinoma cell line and RKO [ATCC number: CRL-2577] colon carcinoma cell line, purchased from Kunming Institution of zoology, were cultured and derivatives were generated. The RKO cells were routinely grown in DMEM/High GLUCOSE (Hyclone, USA) containing 10% Fetal Bovine Serum (FBS; Hyclone, USA) and 1% Penicillin–Streptomycin Solution (PSS, Hyclone, USA), while HCT 116 cells were cultured in RPMI Medium Modified (Hyclone, USA) containing 10% FBS and 1% PSS in accordance with the manufacture’s instruction. After being washed with phosphate-buffered saline (PBS; Hyclone, USA) one or two times, the cells were digested for 2 to 3 min with 0.25% Trypsin (1–2 ml; Gibco). Digestion of RKO cells was ended by FBS-containing medium. Following centrifugation (1000 rpm for 8 min) and re-suspension, cell suspension was collected and the cells were plated in 25T (3 ml) culture flasks at a density of 4 × 10^5^ cells/ml in an incubator. After 24 h (h), the supernatants containing non-adherent cells were removed and fresh medium was added. The cells were passaged after growing approximately to 90% confluency. Subsequently, the HCT-116 and RKO cells were cultivated for further analysis. The growth status of the cultured cells was observed under an inverted microscope (Leica, Germany).

### SCU administration

Firstly, 150 mg SCU (LongJing Biotech, China) were dissolved by 1 ml DMSO into 150 mg/ml and then sterilized by filtration through a 0.22 μm filter. The drug was added to the previously cultured HCT-116 and RKO cells at a set concentration. Normal saline was added into control groups. In each group, cells were observed at 24 h, 48 h, 72 h and 96 h respectively after drug administration to monitor the survival of cells (Additional file 1, 2, 3).

### Cell Counting Kit-8 (CCK-8) Assay

The half maximal inhibitory concentration (IC50) of SCU administration in HCT-116 and RKO cells was evaluated using CCK-8 assays. HCT-116 and RKO cells were plated at a density of 3000 cells per well into 96-well plates in a total volume of 100 μl medium. Then SCU was added into the cells for incubation overnight. At 72 h after dosing, CCK-8 assay was carried out to detect the cell viability. At the end time, CCK-8 solution was added into each well (10 μl per well). Four hours later, optical density (OD) at 450 nm was measured via Thermo Scientific Microplate Reader (Multlskan GO, Thermo) to determine the cell proliferation/viability. All the procedures were performed in triplicate and repeated at least three times. The inhibition ratio of SCU was expressed as a percentage of the control treated with vehicle solutions.

### Terminal-deoxynucleoitidyl transferase mediated nick end labeling (TUNEL) staining

TUNEL staining was employed to detect the apoptosis of HCT-116 and RKO cells. Cells were fixed with 4% paraformaldehyde for 20 min after washed three times with PBS for 5 min each time. Subsequently, being washed three times with PBS again, cells were incubated with a mixture of 0.1% sodium citrate and 0.3% Triton X-100 at 37 °C for 30 min. The TUNEL reaction mixture was prepared in the dark: enzyme solution and label solution in a ratio of 1:9 on ice (In Situ Cell Death Detection Kit, TMR, Roche, USA). The specimens were then put into a dark box to incubate at 37 °C for 1 h, and DAPI containing anti-fluorescence quencher were added to stain the cells which were incubated for 1 min at room temperature. High-content cell imaging system was finally used for picture collection. Positive cells were counted by two researchers blinded to the experiment.

### Flow cytometry analysis

The cell cycles of 1 × 10^6^ HCT-116 and RKO cells in 6-well plates were observed by flow cytometry using a cell cycle analysis kit (PI/RNase Staining Buffer Solution, BD, USA). Briefly, after SCU administration, cells were digested with 0.25% EDTA-free trypsin, followed by centrifugation for 5–10 min at 1000 rpm. After cells collection, cells were re-suspended once with 1× PBS and centrifuged at 1000 rpm for 5–10 min. For cell cycle assay, cells were fixed by pre-chilled 70% ethanol at 4 °C for 18 h. After centrifugation and washes with ethanol to remove ethanol, 500 μl 1× staining solution was added followed by incubating at RT in dark for 15 min. Analysis was performed using a FACSVerse laser flow cytometry analysis system (Becton–Dickinson USA).

### Activity of Caspase3/7

Cultured RKO cells were inoculated with 96-well plates, drug-treated and cultured in a incubator with CO_2_ at 37 °C for 3 days (d). The Caspase-Glo3/7 buffer and the Caspase-Glo3/7 lyophilized powder were placed at room temperature, and Caspase-Glo reaction solution was a mixture of 10 ml Caspase-Glo3/7 buffer and Caspase-Glo3/7 substrate (Caspase-Glo^®^ 3/7Assay, Promega, USA). Afterwards, 100 μl of Caspase-Glo reaction solution was added to each well and shaken on a plate shaker at 300–500 rpm for 30 min. After mixing, the cells were incubated at room temperature for 2 h. Cell status was observed under a fluorescence microscope (Olympus, USA). Data were measured using a microplate reader (Tecan infinite, Switzerland).

### Wound healing test

The wound healing test was performed to detect the migration rate of HCT-116 and RKO cells after SCU administration. Firstly, the number of cells was counted at RT and the cells were prepared into suspension. Then cell suspension was plated at a density of 5 × 10^5^ cells per well in 6-well plates. Next, a linear scratch was performed with a plastic 10 μl pipette tip on the 6-well plates to make sure that the width of the scratches is as equal as possible. Afterwards, PBS was used to wash the cell fragments from scratches. For HCT-116 cells, the images before drug administration at 0 h as well as after drug administration at 12 h and 36 h were captured via Multifunctional automated inverted Fluorescence Microscopy (Nikon Eclipse Ti-S fluorescence microscope, Nikon). Meanwhile, for RKO cells, the images were captured after drug administration at 0 h, 8 h and 24 h. The transferred rate = (the average distance at 0 h − the average distance at × h)/(the average distance at 0 h) × 100%.

### Transwell invasion assay

Transwell invasion assay was employed to investigate the invasion and migration ability of HCT-116 and RKO cells under SCU administration via transwell chamber. Firstly, transwell was placed in a new 24-well plate, and 100 μl of serum-free medium was added to the upper chamber in a 37 °C incubator for 1 h. Cells were suspended in serum-free medium with the specified concentration of drug at a seeding density of 1 × 10^5^/well. After adding 100 μl of cell suspension to the upper chamber, 600 μl of 30% FBS medium was added to the lower chamber. In the invasion test, 500 μl of serum-free medium was added to the upper and lower chambers and placed in a 37 °C incubator for 2 h to rehydrate the Matrigel matrix layer. After adding 500 μl of cell suspension to the upper chamber, 750 μl of 30% FBS medium was added to the lower chamber. Following incubation at 37 °C, non-migrating and non-invading cells in the chamber were removed with a cotton swab, then migrating and invading cells were fixed in 4% paraformaldehyde for 20 min. After being washed with PBS, cells were stained with 600 μl 0.1% crystal violet at RT for 20 min. Following extensive washing with deionized water for several times, the images were captured randomly for at least five fields of each membrane using a fluorescence microscope (DM4000 B, Leica). The number of migrating or invading cells was expressed as the average number of cells per microscopic field over five fields.

### Animals and groups

Forty 4-week-old female nude mice weighing around 18–22 g were provided by Kunming Medical University in SPF environment. Animal Experimental Department) housed in a specific pathogen-free (SPF) were randomly assigned into 5 groups: Control, 5-Fluorouracil (5-FU), Scu-50 mg/kg, Scu-150 mg/kg and Scu-300 mg/kg groups. Animals in negative control group were injected with equivalent DMSO, animals in positive control group were injected with 5-FU, and animals in the other 3 groups were implanted with RKO cell lines (50 mg/kg SCU: 150 mg/kg SCU, and 300 mg/kg SCU). All experimental protocols and animal handling procedures were approved by the Animal Care and Use committee of Kunming Medical University in Yunnan province, China and were consistent with the National Institutes of Health Guide for the Care and Use of Laboratory Animals.

### RKO cells injection and SCU administration in nude mice in vivo

To determine the influence of SCU on RKO cells in vivo, 2E+7/mL RKO cells were subcutaneously implanted into the armpit of the right forelimb of 4-week-old female nude mice, and each animal was injected with 200 μl RKO cells. Then the tumor growth situation was investigated after injection for 5–7 days. When tumors grew to over 100 mm^3^, mice were arranged into 5 groups (control group, 5-FU group, 50 mg/kg, 150 mg/kg, and 300 mg/kg SCU groups, 10 mice in each group). The drug was injected intraperitoneally every other day for a total of 21 days, Control groups were treated with equivalent DMSO, and the mice injected with 5-FU were as positive control group. Experimental mice, 50 mg/kg, 150 mg/kg and 300 mg/kg SCU was intraperitoneally injected into the mice individually every other day for a total of 21 days. The tumor dimensions were measured every 3 d using a digital caliper and the tumor volume was calculated using the following formula: V = π/6 × (Length) × (Width)^2^.

### Positron emission tomography-computed tomography (PET-CT)

PET-CT was performed to investigate the glucose uptake in tumor in nude mice, demonstrated by SUV max. In detail, after injection of RKO cells for 28 days, rats were anesthetized with 0.7% pentobarbital sodium (10 μl/g) via intraperitoneal injection and fixed in the supine position in PET/CT scanning table (Discovery 690/Elite, GE, USA). By the standard protocol of AW Volume Share 5 software for 20 min, the whole-body CT and PET data were acquired. The scanning parameters were set as follows: 120 kV voltage, 260 μA electricity, 0.561 screw pitch, 0.5 s/cycle rotational speed, 3.75 mm thickness and interval, 512 × 512 for CT matrix and fov = 50 cm × 50 cm. Further, CT and PET images were transferred to AW Volume Share 5 workstation to obtain coronal, sagittal, cross-sectional and 3D images, as well as fusion images of CT and PET images. Ultimately, the PET/CT pictures were analyzed through two PET/CT reporters by double blind approach and the average value of SUVmax was confirmed through drawing the ROI of tumor.

### Tissue harvest

After PET-CT protocol, the animals were sacrificed by cervical dislocation following an intraperitoneal injection of 2% pentobarbital sodium (10 μl/g). Then two vernier calipers were placed on the left and the top of the animals to observe the specific scales, and animal pictures were captured by a digital camera. Afterwards, the tumor tissues were removed and the blood was obtained immediately for further analysis of aspartate aminotransferase (AST), alanine transaminase (ALT), white blood cell (WBC), platelet (PLT) and concentration of hemoglobin (HGB). Besides, the tumors were arranged on the whiteboard for picture capturing, and Vernier caliper was also used as a reference to read specific dimensions of tumors. Lastly, the tissues were stored at − 80 °C for further use.

### Microarray analysis in RKO cells with SCU treatment

Affymetrix microarray analysis was performed by KeyGEN BioTECH Shanghai (Shanghai, China) for a ‘whole genome expression profiling gene chip of cells’ test in RKO cells with SCU treatment (n = 4/group). Briefly, total RNA was analyzed by Agilent 2100 and GeneChip 3′IVT Express Kit was used to prepare amplified RNA (aRNA). Following complementary DNA (cDNA) synthesis, double-stranded DNA was synthetized as template. Subsequently, GeneChip 3′IVT Express Kit was used to perform reverse transcription to obtain biotin-labeled cDNA in vitro. The aRNA was purified and fragmented. Afterwards, the fragmented aRNA was hybridized to GeneChip. After hybridization, the GeneChip arrays were eluted and then stained on a GeneChip Fluidics Station 450 followed by scanning on a GeneChip Scanner 3000. The signals and data were acquired and Significant Analysis of Microarray software (SAM) was used to identify significantly differentially expressed genes between the SCU groups and the NC groups. Furthermore, the microarray data was analyzed with Ingenuity Pathway Analysis (IPA).

### Proteomic analysis

Proteomic analysis was employed to investigate the proteome changes in this study. Forty-eight hours following SCU administration, RKO cells were collected with 6 × 10^6^ cells for each sample in SCU group (6 × 10^7^ cells for each one in control group) and there were 4 samples for each group. Protein extraction, digestion, peptide labeling, peptide fractionation, phosphorylated peptides enrichment and mass spectrometer (MS) detection were conducted at Shanghai Luming Biotechnology Co., LTD (Shanghai, China) as described previously [21].

### Statistical analysis

Results used for statistical difference were presented as the mean ± SD. For multiple group comparison, one-way analysis of variance (ANOVA) with Tukey’s post hoc multiple comparisons were applied. And Student’s T test was used to analyze the data between two groups. In addition, nonlinear regression analysis was used to analyze IC50 of SCU in RKO and 116 cells. Ingenuity Pathway Analysis (IPA) was applied for the disease and functions analysis of differentially expressed genes. All statistical analysis was performed using SPSS 13.0 software (SPSS, Inc., Chicago, IL, USA). Significant difference among groups was determined using Bonferroni‑corrected analysis of variance. *p* < 0.05 was considered to indicate a statistical significant difference.

## Results

### The SCU tolerance of RKO and HCT-116 cells

**Fig. 1:**
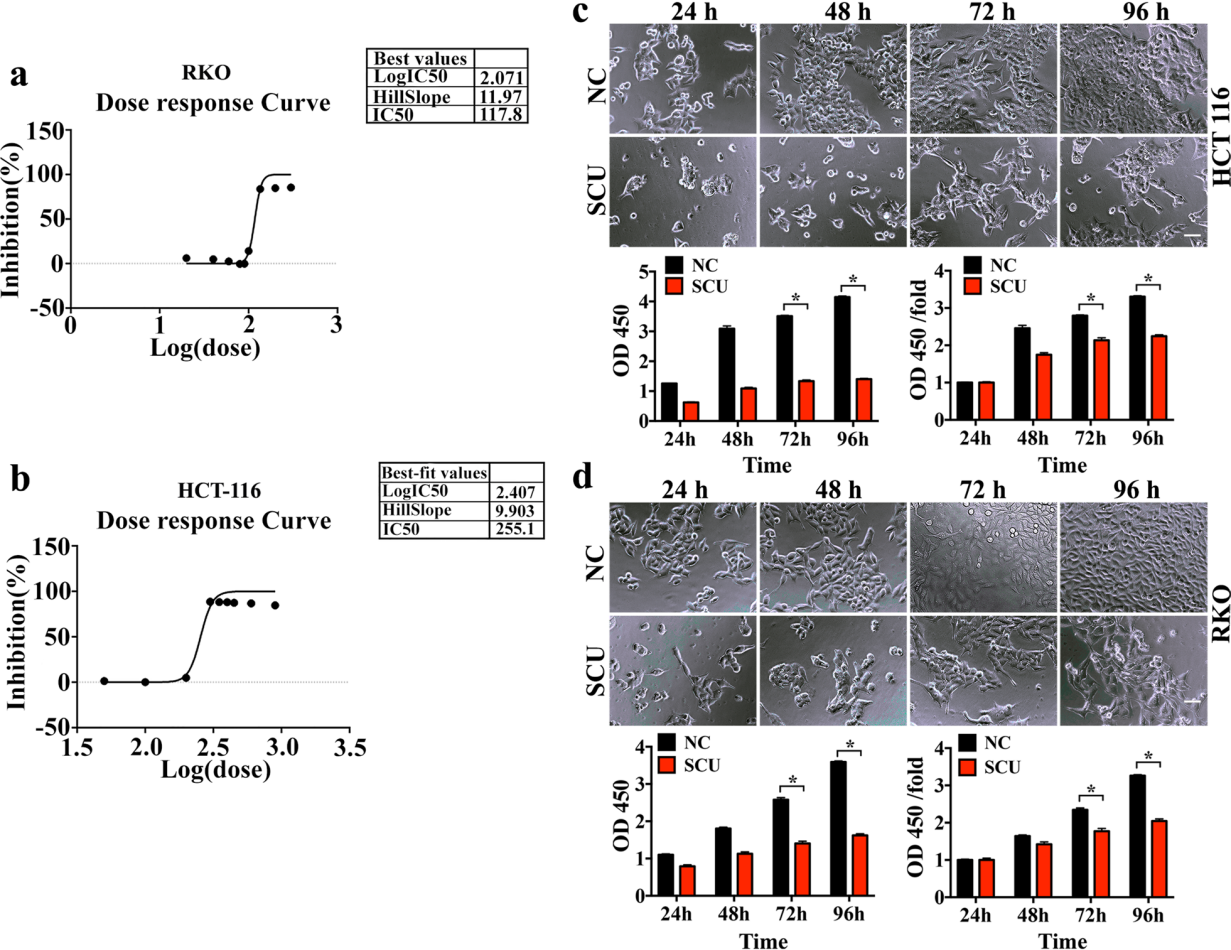
The effects of SCU on cell viability of RKO and HCT-116 cells. **a**, **b** The dose response curve of IC50 from log (0) to log (2) for RKO cells and from log (1.5) to log (3.5) for HCT-116 cells, n = 3. **c**, **d** Cell viability variation of RKO and HCT-116 cells following SCU treatment in NC group and SCU group respectively shown by open field images and line charts of OD value, n = 5. SCU: Scutellarin; NC: negative control; h: hours; IC50: 50% inhibition concentration. All data are analyzed by GraphPad Prism 6 software shown as mean ± SD. Scale bar = 50 μm.*p < 0.05

IC50 of RKO and HCT-116 was detected using CCK-8 assay to determine the sensitivity of these two cell lines in response to SCU treatment. The IC50 of RKO and HCT-116 cells were 117.8 μM and 255.1 μM respectively (Fig. 1a, b). The cell number of RKO and HCT-116 cells showed decrease after being subjected to SCU treatment for 24 h, 48 h, 72 h and 96 h compared to the control groups. Besides, the cellular viability of RKO and HCT-116 cells increased gradually as the culture time prolonged but it was always depressed with the presence of SCU in comparison to control group indicated by OD value at 450 nm, and statistical difference was shown at culturing 72 h and 96 h (Fig. 1c, d, *p* < 0.05). These results indicated that SCU administration resulted in a reduction in the viability of HCT-116 and RKO cell lines.

### SCU suppressed proliferation and induced apoptosis of RKO and HCT-116 cells

**Fig. 2:**
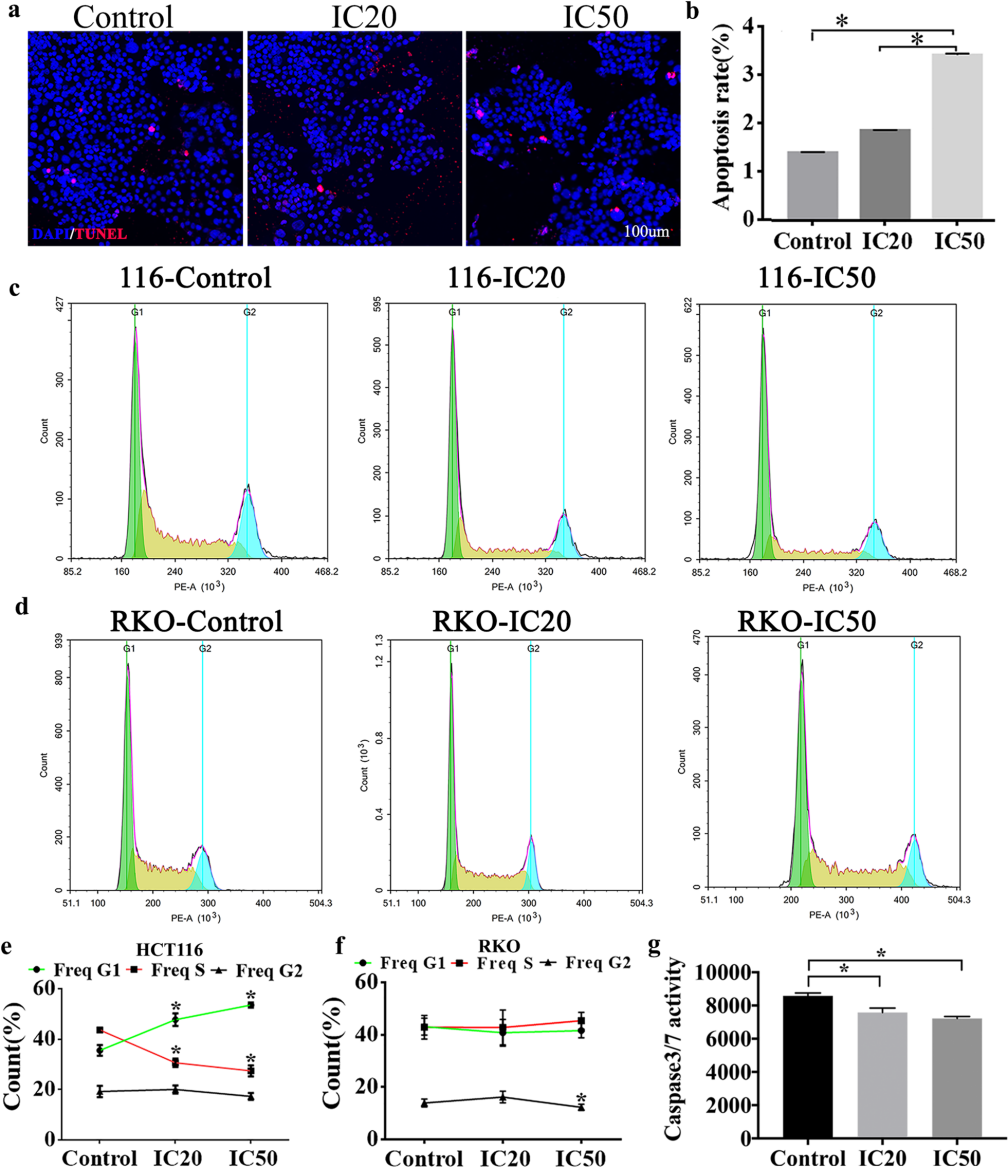
The effects of SCU on the proliferation and apoptosis of HCT-116 and RKO cells. **a** Immunofluorescent staining of TUNEL in Control, IC20 and IC50 groups. Apoptotic cells are stained by red color, and the nucleus is stained by blue. **b** The apoptosis rate (TUNEL/DAPI) comparison among these three groups. The flow cytometry detection of **c** HCT-116 cells and **d** RKO cells in the indicated groups. The cell count variation of **e** HCT-116 cells and **f** RKO cells in Control, IC20 and IC50 groups. **g** The variation of Caspase3/7 activity in control, IC20 and IC50 groups. IC20: 20% inhibition concentration; IC50, 50% inhibition concentration; SCU, Scutellarin; TUNEL, Terminal-deoxynucleoitidyl Transferase Mediated Nick End Labeling; DAPI, 4′,6-diamidino-2-phenylindole. All data are shown as mean ± SD, n = 5. *p < 0.05. Scale bar = 100 μm

To further validate the cell viability of HCT-116 cells after SCU treatment, immunofluorescent staining for TUNEL staining was applied. As a result, the number of apoptotic positive HCT-116 cells significantly increased in IC20 group and IC50 group, especially in IC50 group compared to control group (Fig. 2a) and apoptosis rate of HCT-116 cells was markedly elevated following SCU treatment with different doses, of which IC50 was more effective than IC20 (Fig. 2b, *p* < 0.05). Besides, flow cytometry was employed to further analyze the cell cycle of HCT-116 and RKO cells treated with different concentrations of SCU (Fig. 2c, d). The outcomes revealed that in HCT-116 cells, the proportion of Freq G1 cells in the IC20 group in HCT-116 cells was significantly increased,while Freq S was decreased. At the same time, this change was more significant in the IC50 group (Fig. 2e, *p* < 0.05). Moreover, in RKO cells, the proportion of Freq G2 cells in the IC50 group was reduced compared to the control group (Fig. 2f, *p *< 0.05). These results suggested that SCU depressed the growth of HCT-116 and RKO cells, prompting apoptosis of HCT-116 and RKO cells, of which IC50 was more effective than IC20 in facilitating cells apoptosis. The pro-apoptotic effects of SCU was further verified by the decreased Caspase 3/7 activity in IC20 and IC50 group relative to that in control group (Fig. 2g, *p* < 0.05).

### The migration of HCT-116 and RKO cells was inhibited after SCU administration in vitro

**Fig. 3:**
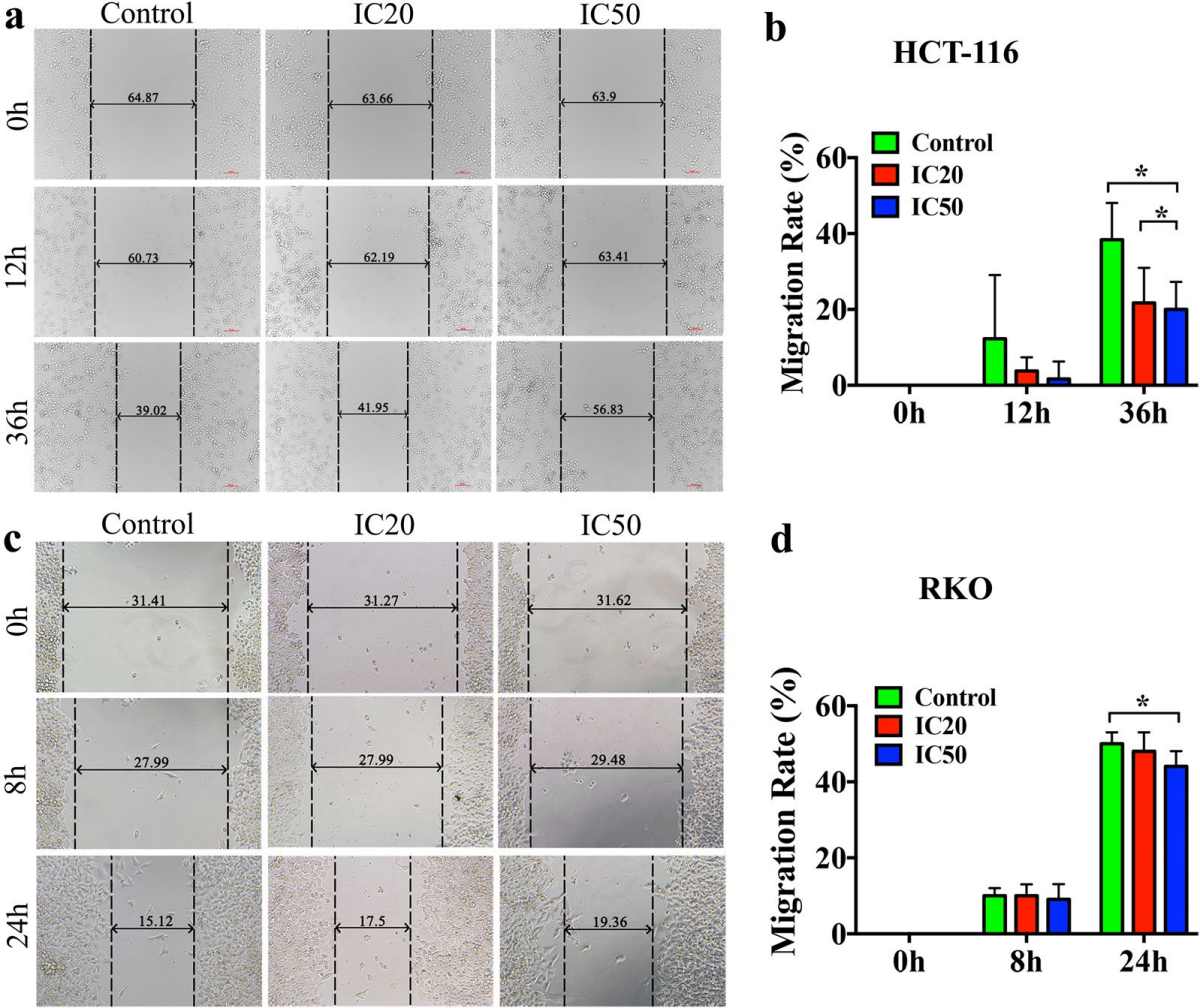
The effects of SCU on migration rate of HCT-116 and RKO cell lines. The cell migration was measured by wound healing assays in **a** HCT-116 cells at 0 h, 12 h and 36 h, and **c** RKO cells at 0 h, 8 h and 24 h. Scale bar = 100 μm. **b** The quantitative analysis of the migration rate of HCT-116 cells and **d** RKO cells. SCU, Scutellarin; IC20, 20% inhibition concentration; IC50, 50% inhibition concentration; h, hours. All data are shown as mean ± SD, n = 5. *p < 0.05

The migration rate of HCT-116 and RKO cells was detected by wound healing assays. The results showed that the cell migration ability of HCT-116 and RKO cells were decreased after SCU treatment (Fig. 3a, c). As time prolonged, cell migration of the SCU-treated HCT-116 cells was inhibited in a dose-dependent manner, and the migration rate was significantly decreased in IC20 and IC50 groups at 36 h compared to the control group (Fig. 3a, b, *p* < 0.05). Similar to HCT-116 cells, cell migration of RKO cells after SCU treatment was obviously suppressed and its migration rate was substantially reduced in IC50 group at 24 h (Fig. 3c, d, *p* < 0.05). All the findings suggested that SCU administration could reduce the migration rate of tumor cells.

### Cellular metastasis of HCT-116 and RKO cells was attenuated after SCU administration

**Fig. 4:**
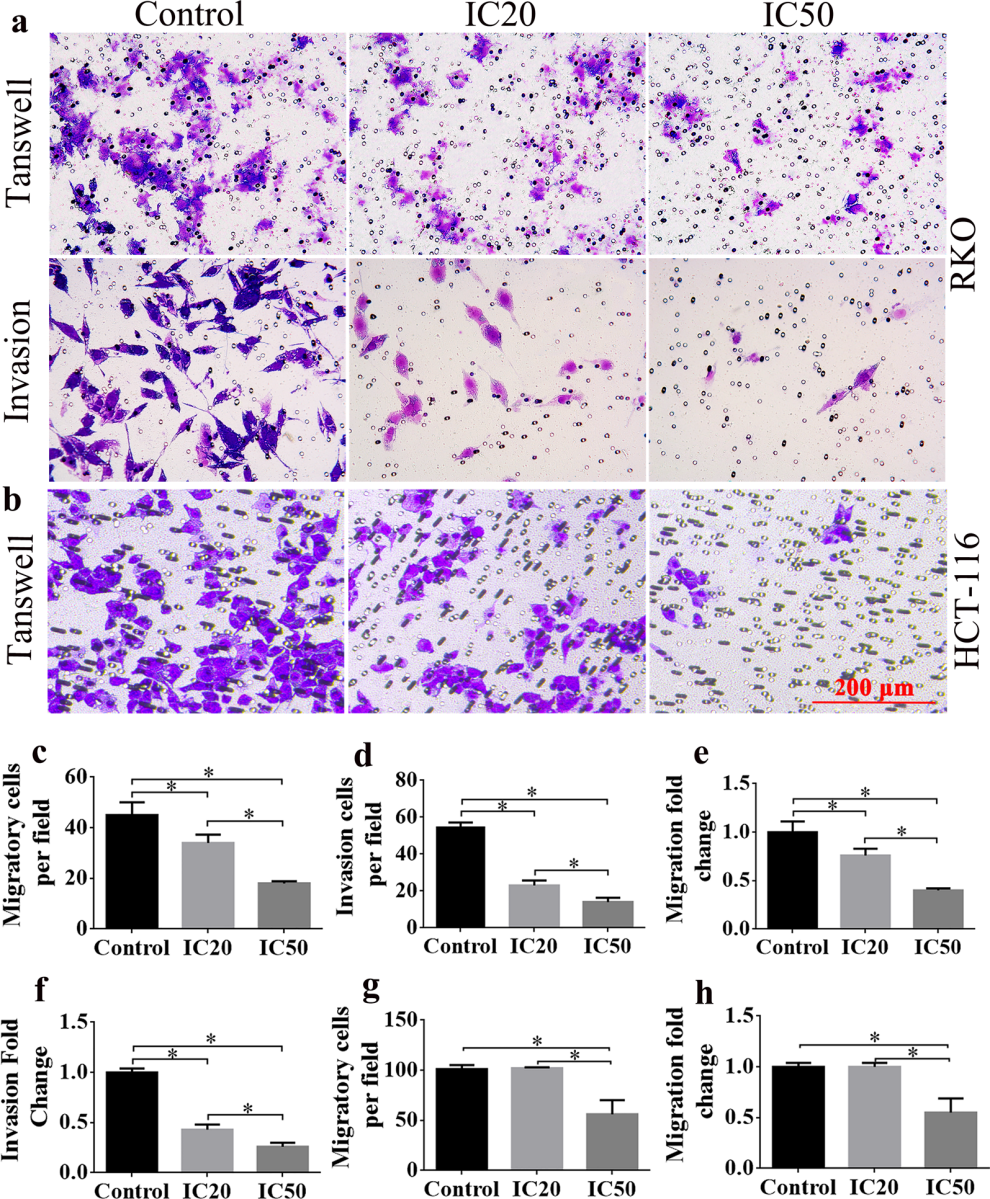
The effects of SCU on the migration and invasion capacities of HCT-116 and RKO cells. The cell migration and invasion in **a** RKO cells and **b** HCT-116 cells were analyzed by transwell assay. Scale bar = 50 μm. **c**, **d** The migratory cells per field and invasion cells per field of RKO cells in control, IC20 and IC50 groups. **e**, **f** The migration fold change and invasion fold change of RKO cells. **g**, **h** The migratory cells per field and invasion cells per field of HCT-116 cells. SCU, Scutellarin; IC20, 20% inhibition concentration; IC50, 50% inhibition concentration. All data are shown as mean ± SD, n = 5. *p < 0.05

To assess the effect of SCU on CRC cell metastasis, HCT-116 and RKO cells were planted in vitro and transwell invasion assay was performed. As the results demonstrated, the migration and invasion ability of RKO and HCT-116 cells were significantly decreased in a dose-dependent manner (Fig. 4a, b). It was exhibited that migratory cells per field of RKO cells in IC50 group were significantly fewer than IC20 and control groups, and migration fold change in IC50 group was much smaller than IC20 and control groups (Fig. 4c, e, *p* < 0.05). Similar trend was shown in invasion cells per well of RKO cells and invasion fold change (Fig. 4d, f, *p* < 0.05). Furthermore, the migratory cells per field and migratory fold change of HCT-116 cells in IC50 group were notably reduced compared to IC20 and control groups (Fig. 4g, h, *p* < 0.05). All of these exhibited that SCU possessed the ability to depress the migration and invasion ability of CRC cells.

### SCU treatment inhibited the tumor growth in nude mice in vivo

**Fig. 5:**
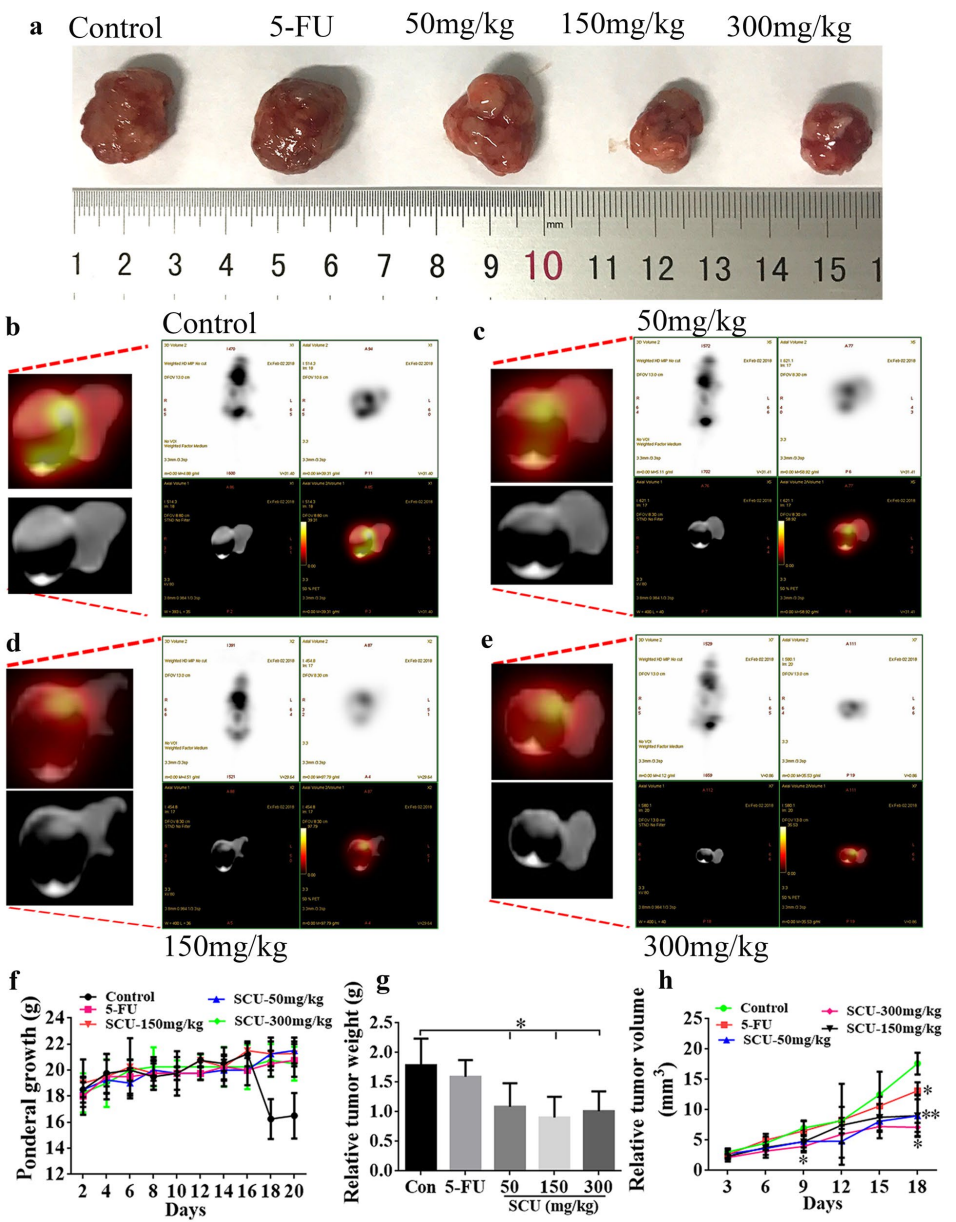
The inhibitory effect of SCU administration on tumor growth in nude mice in vivo. **a** The morphology of tumor in nude mice after the administration of DMSO (control) and 25 mg/kg 5-FU as well as 50 mg/kg, 150 mg/kg and 300 mg/kg SCU. Scale bar = 1 cm. PET-CT assay of tumor size in **b** control, **c** Scu-50 mg/kg, **d** Scu-150 mg/kg and **e** Scu-300 mg/kg groups. **f** Variation of ponderal growth in control, 5-FU, Scu-50 mg/kg, Scu-50 mg/kg and Scu-50 mg/kg groups from 2 d to 20 days. **g** The relative weight of tumor in nude mice after the administration of DMSO (control) and 25 mg/kg 5-FU as well as SCU with 50 mg/kg, 150 mg/kg and 300 mg/kg. **h** The relative volume changes of tumor after the administration of DMSO (control) and 25 mg/kg 5-FU as well as SCU with 50 mg/kg, 150 mg/kg and 300 mg/kg at 3, 6, 9, 12, 15, 18 d. Con: Control; 5-FU: 5-Fluorouracil; SCU, Scutellarin; d, day. All data are shown as mean ± SD, n = 10, *p < 0.05; **p < 0.01

With the intent to investigate the effect of SCU on tumor growth, the RKO cells were injected into the nude mice, and different doses of SCU were tested for the treatment of tumor in nude mice in vivo. It was demonstrated that 50 mg/kg, 150 mg/kg and 300 mg/kg SCU significantly decreased the tumor weight compared with control group (Fig. 5a, g, *p* < 0.05). Additionally, PET-CT was carried out to detect the glucose uptake of the tumor, quantified by SUV max. As a result, SCU administration induced a decreased glucose uptake in the tumor in a dose-dependent manner, of which 300 mg/kg SCU showed the lowest glucose uptake (Fig. 5b–e). Nevertheless, no significant difference was exhibited in ponderal growth (Fig. 5f). Further, the tumor volume was smaller after 5-FU treatment at 18 d than that of control group (*p* < 0.01), 150 mg/kg SCU administration obviously reduced the relative tumor volume at 18 days (*p* < 0.01), and 300 mg/kg SCU administration markedly decreased the tumor volume at 9, and 18 days compared with control group (Fig. 5h, *p* < 0.05).

### Microarray analysis of differentially expressed genes in RKO cells after SCU administration

**Fig. 6:**
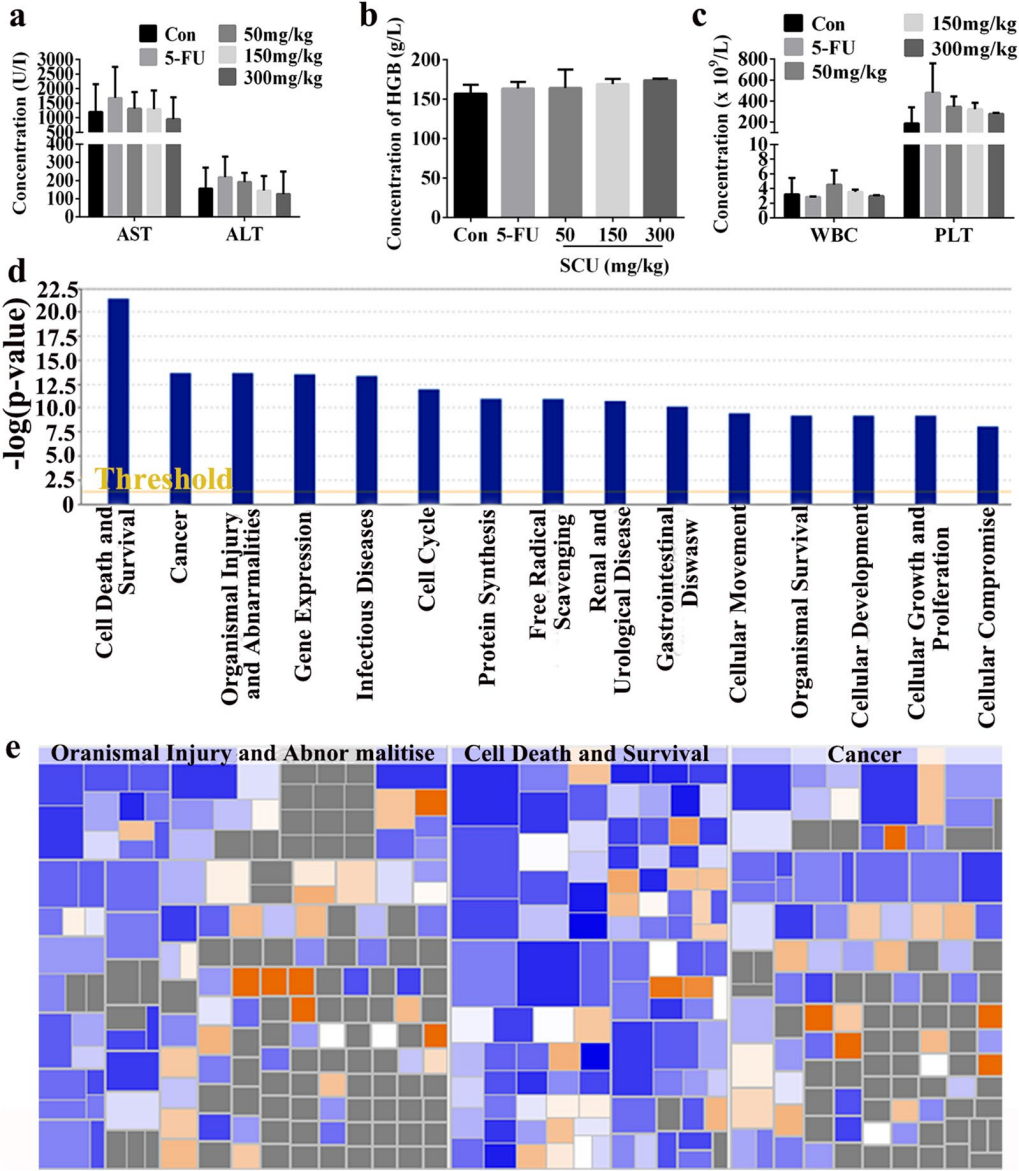
Ingenuity pathway analysis of genes function in RKO cells. The concentration of **a** AST and ALT, **b** HGB as well as **c** WBC and PLT in control, 5-FU as well as SCU with 50 mg/kg, 150 mg/kg and 300 mg/kg. All data are shown as mean ± SD, n = 4/group. **d** Disease and function bar graph of differentially expressed genes in classification of diseases. **e** The heat maps of relationship between upregulated and downregulated genes in activation or inhibition of diseases. The orange stands for z-score > 0; the blue stands for z-score < 0; the gray means no Z-score value. *Con * control, *5-FU* 5-fluorouracil, *Scu* scutellarin, *d* day, *HGB* hemoglobin, *AST* aspartate aminotransferase, *ALT* alanine transaminase, *WBC* white blood cell, *PLT* platelet

Blood routine and biochemistry examination were performed to verify whether there were side effects after SCU administration. As the results showed, the concentration of HGB, AST, ALT, WBC and PLT was detected after SCU administration, however, no significant changes were revealed among 50 mg/kg, 150 mg/kg and 300 mg/kg SCU addition in HGB, AST, ALT, WBC and PLT concentration test (Fig. 6a–c). In order to investigate the underlying mechanisms of SCU treatment for CRC, Microarray analysis was performed to provide insights into the disease and functional classifications in which differentially expressed genes involved. It was demonstrated the differentially expressed genes were mainly enriched in these classifications including cell death and survival, cancer, organismal injury and abnormalities, gene expression, protein synthesis, free radical scavenging, renal and urological disease, cellular movement, organismal survival, cellular development, cellular growth and proliferation and cellular compromise (Fig. 6d). Additionally, the heat map of disease and functions revealed that these three functions, cell death and survival, cancer, organismal injury and abnormalities were significantly suppressed by differentially expressed genes (Fig. 6e).

### Functional clustering analysis of differentially expressed proteins in SCU-administered RKO cells

**Fig. 7:**
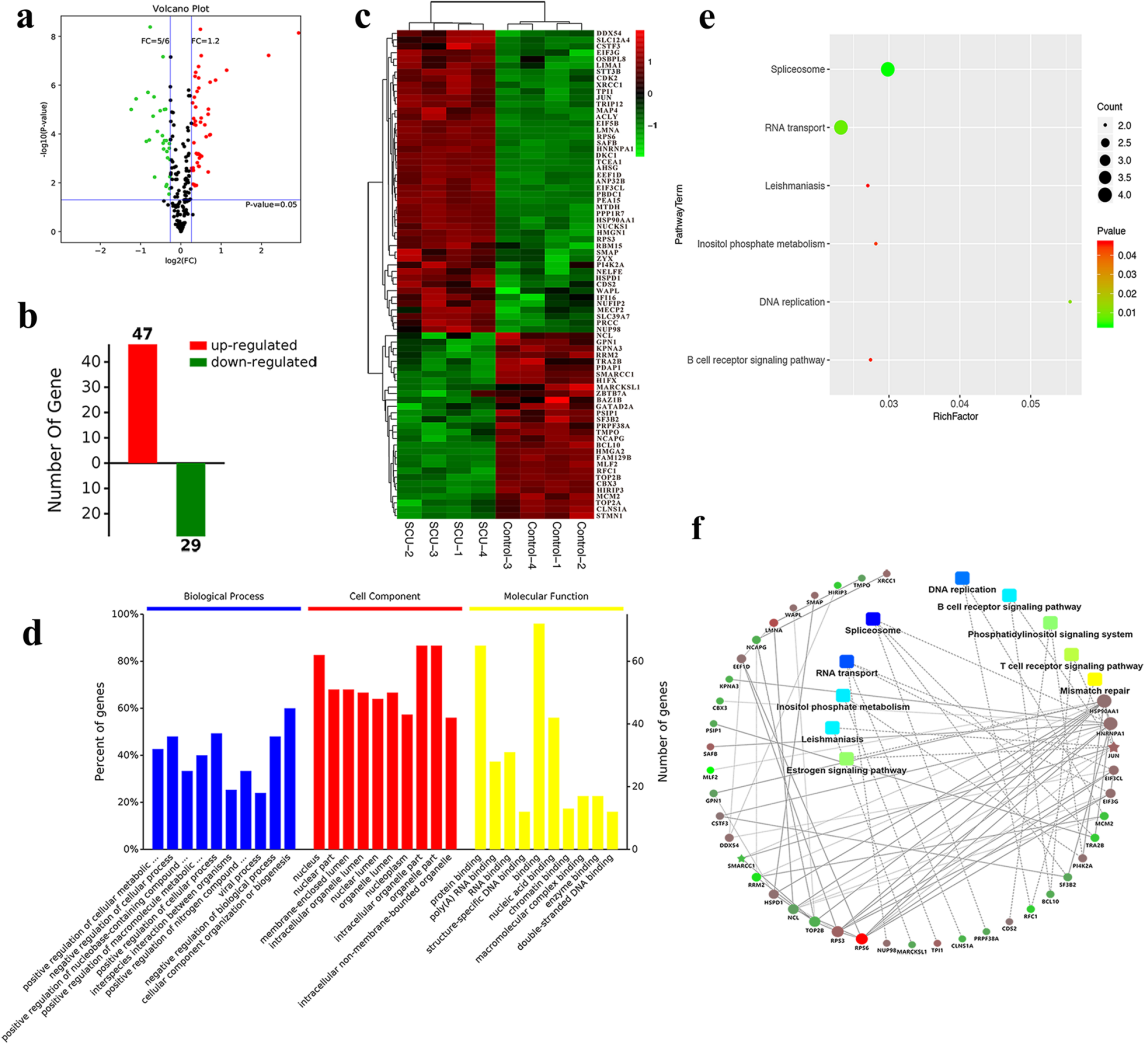
Proteomic analysis of differentially expressed proteins. **a** Differentially expressed proteins shown by volcano plot. Fold change > 1.2 or < 5/6 and P < 0.05 is considered to be a significant differentially expressed protein. Red for up-regulated proteins, green for down-regulated ones, and black for no differentially expressed proteins. **b** Number of identified up- or down regulated proteins. **c** Heat maps of identified proteins in control and SCU groups. **d** GO analysis of DEPs’ biological functions. **e** Statistics of KEGG pathway enrichment of DEPs. Rich Factor is the ratio of DEP number annotated in this pathway term to all protein number annotated in this pathway term. Greater Rich Factor means greater effect of the inhibitors on the analyzed pathway. **f** Red dot represents upregulated protein, green for down-regulated one. Rectangles represent biological processes, cellular localization, molecular functions or signaling pathways. Blue for higher P value while yellow for the lower. Solid lines represent protein (genes)-proteins (genes) are interrelated, and dashed lines represent metabolic pathways-proteins (genes) are interrelated. All data are shown as mean ± SD, n = 4. *SCU * scutellarin

By proteomic analysis of RKO cells in the NC group and SCU group, the Volcano plot exhibited differentially expressed proteins (DEPs), red for up-regulated proteins, green for down-regulated ones, and black for proteins without differential expression and further identified 47 upregulated proteins and 29 downregulated proteins with significant difference (Fig. 7a, b). Additionally, the clustering analysis demonstrated the expression variation of each protein identified above in SCU and Control groups (Fig. 7c). Functional annotation of all the identified proteins was conducted based on the annotation information from the Gene Ontology (GO) database and the Kyoto Encyclopedia of Genes and Genomes (KEGG) database (Fig. 7d). According to the enrichment factor, the top 10 biological processes was selected: the positive regulation of cellular metabolic, negative regulation of cellular process, positive regulation of nucleobase-containing compound, positive regulation of macromolecule metabolic, positive regulation of cellular process, interspecies interaction between organisms, positive regulation of nitrogen compound, viral process, negative regulation of biological process and cellular component organization or biogenesis. In accordance with enrichment factor, the top 10 cell components were: nucleus, nucleus part, membrane-enclosed lumen, intracellular organelle lumen, nuclear lumen, nucleoplasm, intracellular organelle part, organelle part and intracellular non-membrane-bounded organelle. The top 10 molecular functions according to enrichment factor were: protein binding, poly(A) RNA binding, RNA binding, structure-specific DNA binding, binding, nucleic acid binding, chromatin binding, macromolecular complex binding, enzyme binding and double-stranded DNA binding (Fig. 7d).

Pathway enrichment analysis of the differentially expressed proteins was also conducted based on the KEGG database in order to explore the changes of metabolic pathways. The top 6 pathways related to spliceosome, RNA transport, leishmaniasis, insolital phosphate metabolism, DNA replication and B cell receptor signaling pathway were significantly enriched (Fig. 7e). Interestingly, spliceosome showed significant interactions with downregulated proteins like PRPF38A, SF3B2 and TRA2B, and RNA transport exhibited marked relation with downregulated protein–CLNS1A. DNA replication was significantly interacted with downregulated proteins, RFC1 and MCM2, and B cell receptor signaling pathway was interacted with downregulated proteins BCL10 and JUN (without differential expression). Meanwhile, JUN was significantly interacted with downregulated protein—SMARCC1 (Fig. 7f).

## Discussion

Here, we reported that SCU was capable of inhibiting the proliferation, invasion and migration, as well as inducing the apoptosis of HCT-116 and RKO cells by comprehensive in vitro experiments. Additionally, experiments performed in nude mice validated that SCU could reduce energy absorption and inhibit the growth of CRC with no side effects in the blood and liver system. Moreover, the underlying mechanisms of SCU’s efficacy on CRC were investigated in this study by applying genomic and proteomic analysis, which could provide theoretical basis for the further clinical treatment for CRC.

CRC, with poor prognosis, has been considered a potentially deadly disease on account for its undefined metastasis in a majority of patients [22]. A large number of evidence showed that researchers’ attention has been drawn to TCM treatment as a result of few side effects and multi-component therapeutic functions [23]. SCU, previously reported to exhibit anti-inflammatory and anti-apoptotic properties and decreases blood pressure in hypertensive rats [24], has been widely studied in the field of carcinoma, for example, Burkitt lymphoma [25], tongue carcinoma [26] and hepatocellular carcinoma [27]. SCU was also demonstrated to be capable of inhibiting the proliferation and induce the apoptosis of cancerous cells. In our present study, TUNEL staining, and Caspase 3/7 activity, flow cytometry analysis, and the wound healing, transwell invasion assays revealed that SCU suppressed the proliferation, invasion as well as migration, and facilitated the apoptosis of HCT-116 and RKO cells. Meanwhile, SCU exhibited abilities to decrease the energy absorption and the growth and volume of CRC tumor in nude mice reflected by PET-CT and in vivo imaging. What’ more, it’s shown that SCU exert no significant adverse effect on weight gain in mice and blood serum biochemical indices. The present study has verified the possible anti-cancer drug for inhibition of tumor growth and migration in the nude mice model with CRC.

In this study, Affymetrix microarray analysis revealed the enrichment of differentially expressed genes in RKO cells after SCU administration in disease and functional classifications and the top 15 classifications were shown as follows: cell death and survival, cancer, organismal injury and abnormalities, gene expression, protein synthesis, free radical scavenging, renal and urological disease, cellular movement, organismal survival, cellular development, cellular growth and proliferation and cellular compromise. Among these disease and functions, organismal injury and abnormalities, cell death and survival, and cancer were prominently inhibited. As a prevalent natural medicine herb, SCU has been used to treat ischemia heart diseases, neurological disorders, hepatitis, inflammation and osteomyelitis [28], and exhibited potent effects in inhibiting the growth of colon cancer, tongue carcinoma and squamous cell carcinoma [26, 29].

Through proteomic analysis, we further identified 47 upregulated proteins and 29 downregulated proteins in the SCU-treated RKO cells, indicating the effect of SCU treatment in colorectal cancer was a multiple-gene regulated process. As revealed by bioinformatics analysis, the functions of the differentially expressed proteins were involved in various biological processes, cellular component and molecular function and mounting correlated signal pathways. Biological function analysis revealed that the identified proteins were mainly involved in the positive regulation of cellular metabolic, negative regulation of cellular process, positive regulation of nucleobase-containing compound, positive regulation of macromolecule metabolic, positive regulation of cellular process, interspecies interaction between organisms, positive regulation of nitrogen compound, viral process, negative regulation of biological process and cellular component organization or biogenesis, suggesting that SCU may play an important role in negatively mediating cellular process of tumor cells but the further investigations are still required. SCU has been previously reported to produce many biological activities, including anti‑oxidative, anti‑inflammatory, cardioprotective effects, as well as effects against human immunodeficiency virus [30, 31]. Combined with our findings, the effects of SCU for the treatment of colorectal cancer were regulated by the interacting effects of various genes involved in the mediation of multiple systems.

Besides, the cellular analysis elucidated the differentially expressed proteins were mainly contained in nucleus, nucleus part, membrane-enclosed lumen, intracellular organelle lumen, nuclear lumen, nucleoplasm, intracellular organelle part, organelle part and intracellular non-membrane-bounded organelle. In addition, molecular function analysis concealed that the differentially expressed proteins mainly participated in protein binding, poly(A) RNA binding, RNA binding, structure-specific DNA binding, binding, nucleic acid binding, chromatin binding, macromolecular complex binding, enzyme binding and double-stranded DNA binding. SCU could inhibit cell proliferation and induce apoptosis in colorectal cancer, accompanied by triggering G2/M phase cell cycle arrest [32]. It has also been demonstrated that SCU epigenetic could modulate DNA, including DNA promoter methylation and histone modification which caused varied expression paterns of miRNA [33]. Meanwhile, pathway enrichment analysis of the DEPs explored the changes of metabolic pathways. The top 6 Pathways were as follows: spliceosome, RNA transport, leishmaniasis, insolital phosphate metabolism, DNA replication and B cell receptor signaling pathway were significantly enriched. Importantly, protein–protein interactions analysis uncovered significant interactions between spliceosome and downregulated proteins like PRPF38A, SF3B2 and TRA2B, between RNA transport and downregulated protein–CLNS1A, between DNA replication and downregulated proteins, RFC1 and MCM2, between B cell receptor signaling pathway and downregulated protein BCL10 and JUN. Meanwhile, JUN was significantly interacted with downregulated protein—SMARCC1.

## Conclusions

Collectively, the findings of the present study identified that Scutellarin could present anticancer activities in colorectal cancer. The present study may provide a new therapeutic strategy for colorectal cancer and may provide data resource based on the genomic and proteomic analysis, therefore act as a guide for future basic research and clinical research.

## Supplementary information

**Additional file 1.** The original IC50 data of RKO cells calculated by Graphpad Prism.

**Additional file 2.** The original IC50 data of HCT-116 cells calculated by Graphpad Prism.

**Additional file 3.** The original data about drug inhibition rate in RKO and HCT-116 cells.

## Data Availability

The datasets analyzed during the current study available from the corresponding author on reasonable request.

## Abbreviations

SCU: Scutellarin
NC: Negative control
IC50: 50% inhibition concentration
EdU: 5-Ethynyl-2′-deoxyuridine
TUNEL: Terminal-deoxynucleoitidyl transferase mediated nick end labeling
DAPI: 4′,6-Diamidino-2-phenylindole
5-FU: 5-Fluorouracil
HGB: Hemoglobin
AST: Aspartate aminotransferase
ALT: Alanine transaminase
WBC: White blood cell
PLT: Platelet
h: Hours

## References

[1] Hu XQ (2018). Plasma metabolic profiling on postoperative colorectal cancer patients with different traditional Chinese medicine syndromes. Compl Ther Med.

[2] Yang DH (2015). Recent advances in understanding colorectal cancer and dysplasia related to ulcerative colitis. Korean J Gastroenterol.

[3] Shaw E (2018). Effects of physical activity on colorectal cancer risk among family history and body mass index subgroups: a systematic review and meta-analysis. BMC Cancer.

[4] Butterworth AS, Higgins JPT, Paul P (2006). Relative and absolute risk of colorectal cancer for individuals with a family history: a meta-analysis. Eur J Cancer.

[5] Taylor DP (2010). Population-based family history-specific risks for colorectal cancer: a constellation approach. Gastroenterology.

[6] Siegel R, Naishadham D, Jemal A (2013). Cancer statistics, 2013. Cancer J Clin.

[7] Virgo KS (1996). Cost of patient follow-up after potentially curative lung cancer treatment. JAMA.

[8] Peeters CFJM (2010). Outgrowth of human liver metastases after resection of the primary colorectal tumor: a shift in the balance between apoptosis and proliferation. Int J Cancer.

[9] Lange PH (1980). Acclerated growth of testicular cancer after cytoreductive surgery. Cancer.

[10] Torre LA (2015). Global cancer statistics, 2012. CA Cancer J Clin.

[11] Li W (2016). Therapeutic targets of traditional chinese medicine for colorectal cancer. J Tradit Chin Med.

[12] Hu XQ (2016). Advances in synergistic combinations of Chinese herbal medicine for the treatment of cancer. Curr Cancer Drug Targets.

[13] Li-Li L (2007). Protective effects of scutellarin and breviscapine on brain and heart ischemia in rats. J Cardiovasc Pharmacol.

[14] Thao T (2014). Two new neoclerodane diterpenoids from *Scutellaria barbata* D. Don growing in Vietnam. J Asian Nat Prod Res.

[15] Zhang L (2014). Chloroform fraction of *Scutellaria barbata* D. Don promotes apoptosis and suppresses proliferation in human colon cancer cells. Mol Med Rep.

[16] Hou L, Chen L, Fang L (2017). Scutellarin inhibits proliferation, invasion, and tumorigenicity in human breast cancer cells by regulating HIPPO-YAP signaling pathway. Med Sci Monit.

[17] Xie Z (2019). Scutellarin synergistically enhances cisplatin effect against ovarian cancer cells through enhancing the ability of cisplatin binding to DNA. Eur J Pharmacol.

[18] Deng W (2018). Scutellarin inhibits human renal cancer cell proliferation and migration via upregulation of PTEN. Biomed Pharmacother.

[19] Yang N (2017). Scutellarin suppresses growth and causes apoptosis of human colorectal cancer cells by regulating the p53 pathway. Mol Med Reports.

[20] Zhu PT (2017). Scutellarin suppresses human colorectal cancer metastasis and angiogenesis by targeting ephrinb2. Am J Transl Res.

[21] Li P (2019). Proteomic profiling and integrated analysis with transcriptomic data bring new insights in the stress responses of Kluyveromyces marxianus after an arrest during high-temperature ethanol fermentation. Biotechnol Biofuels.

[22] Jin Y (2017). Scutellaria barbata D. Don inhibits migration and invasion of colorectal cancer cells via suppression of PI3K/AKT and TGF-beta/Smad signaling pathways. Exp Ther Med.

[23] Shen AL (2012). Effects of Pien Tze Huang on angiogenesis in vivo and in vitro. Chin J Integr Med.

[24] Chen X (2013). Scutellarin attenuates hypertension-induced expression of brain Toll-like receptor 4/nuclear factor kappa B. Mediat Inflamm.

[25] Feng Y (2012). Novel function of scutellarin in inhibiting cell proliferation and inducing cell apoptosis of human Burkitt lymphoma Namalwa cells. Leuk Lymphoma.

[26] Li H (2010). Scutellarin inhibits cell migration by regulating production of alphavbeta6 integrin and E-cadherin in human tongue cancer cells. Oncol Rep.

[27] Xu H, Zhang S (2013). Scutellarin-induced apoptosis in HepG2 hepatocellular carcinoma cells via a STAT3 pathway. Phytother Res.

[28] Kim DI (2005). Regulation of IGF-I production and proliferation of human leiomyomal smooth muscle cells by *Scutellaria barbata* D Don in vitro: isolation of flavonoids of apigenin and luteolin as acting compounds.. Toxicol Appl Pharmacol.

[29] Dai ZJ, Wang XJ, Li ZF, Ji ZZ, Ren HT, Tang W, Liu XX, Kang HF, Guan HT, Song LQ (2008). Scutellaria barbate extract induces apoptosis of hepatoma H22 cells via the mitochondrial pathway involving caspase-3. WJG..

[30] Hong H, Liu GQ (2004). Protection against hydrogen peroxide-induced cytotoxicity in PC12 cells by scutellarin. Life Sci.

[31] Pan Z, Zhao W, Zhang X, Wang B, Wang J, Sun X, Liu X, Feng S, Yang B, Lu Y (2011). Scutellarin alleviates interstitial fibrosis and cardiac dysfunction of infarct rats by inhibiting TGFβ1 expression and activation of p38-MAPK and ERK1/2. Br J Pharmacol.

[32] Gao C, Zhang H, Guo Z, You T, Chen X, Zhong D (2012). Mechanistic studies on the absorption and disposition of scutellarin in humans: selective OATP2B1-mediated hepatic uptake is a likely key determinant for its unique pharmacokinetic characteristics. Drug Metab Dispos.

[33] Yang B, Zhao YL, Yang X, Liao XL, Yang J, Zhang JH, Gao CZ (2013). Scutellarin-cyclodextrin conjugates: synthesis, characterization and anticancer activity. Carbohyd Polym.

